# Reply to Letter to the Editor

**DOI:** 10.3906/sag-1909-140

**Published:** 2020-02-13

**Authors:** Zehra İpek ARSLAN

**Affiliations:** 1 Department of Anesthesiology and Reanimation, Faculty of Medicine, Kocaeli University, Kocaeli Turkey

**To the Editor,**

On behalf of my coauthor, I would like to thank Dr. Shao and colleagues for their interest and comments posing the question ‘Does selection of nostrils really affect performance of nasotracheal intubation with nasotracheal Airtraq®?’ on our manuscript, ‘Which nostril should be used for nasotracheal intubation with Airtraq NTÒ: the right or left? A randomized clinical trial’, which recently appeared in the Turkish Journal of Medical Sciences [1]. 

The results of our study showed that nasotracheal intubation via the right nostril could be performed in a shorter time when compared to the left nostril with the Airtraq NT. When we performed another trial that compared video laryngoscopes for nasotracheal intubation, we realized that there was a significant difference between the right and the left nostril in patients in the Airtraq NT group. Therefore, we decided to perform this trial comparing the right and the left nostrils during nasotracheal intubation with the Airtraq NT. Our answer to their question is ‘Yes, nasotracheal intubation was easier through the right nostril while intubating with the Airtraq NT without using Magill forceps’. The reason for this result was the removal of the channel from the right side of the nasotracheal Airtraq when compared to the standard Airtraq. However, the mirror and the camera were present on the left side of the Airtraq NT. As a result, there was much more space at the right nasopharyngeal side. This allows for easier manipulation of the endotracheal tube. 

There was a published multiplanar magnetic resonance imaging study that exhibited the association of the ease of nasotracheal intubation through the right nostril and the posterior pharyngeal anatomy even with direct laryngoscopy [2]. In addition, there are published studies that showed intubation to be easier from the right nostril even with the aid of Magill forceps and direct laryngoscopy [3,4]. There is a published case of nasotracheal intubation of a difficult airway with oral cancer, which was performed easily with a nasotracheal Airtraq through the right nostril without using Magill forceps [5]. 

## Key words: 

Airtraq, nasotracheal, right nostril, video laryngoscope
